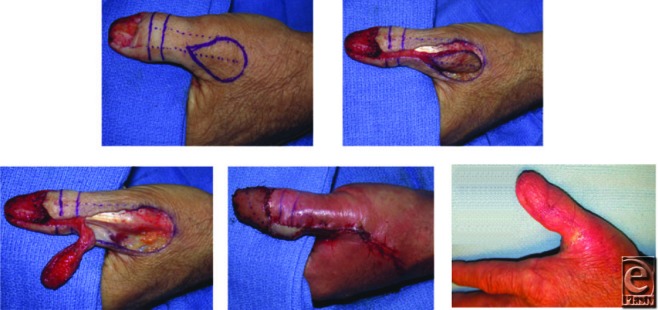# Reconstruction of a Distal Dorsal Thumb Defect

**Published:** 2013-04-17

**Authors:** Lily Daniali, Kodi Azari

**Affiliations:** ^a^Department of Surgery, Division of Plastic Surgery, New Jersey Medical School, University of Medicine and Dentistry of New Jersey; ^b^Department of Orthopaedic Surgery/Orthopaedic Hospital and Division of Plastic Surgery, David Geffen School of Medicine at University of California Los Angeles

**Figure:**
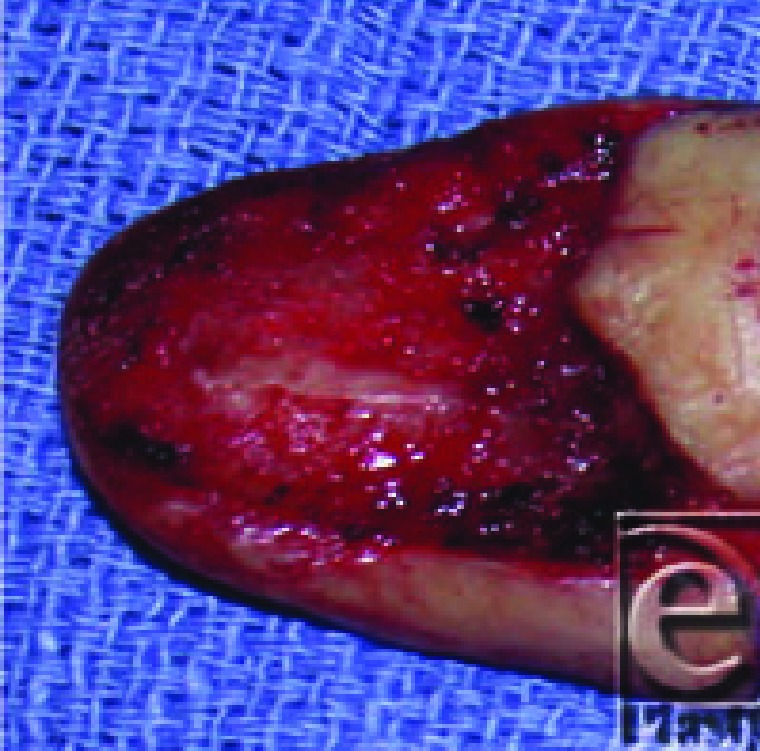


## DESCRIPTION

A 61 year-old man was referred for management of a distal dorsal squamous cell carcinoma of the thumb with involved distal phalangeal periosteum. The dorsal cortex of the distal phalanx and all nail-producing and hyponychial tissues were excised and sent for pathology. No tumor was found; thus, the remaining volar distal phalanx was conserved.

## QUESTIONS

1. **Are there any unique reconstructive goals particular to thumb reconstruction?**
2. **What is the arterial supply to the thumb?**
3. **What reconstructive options are available for the reconstruction of a dorsal thumb defect?**

## DISCUSSION

The core plastic surgery tenet of replacing “like with like” holds particularly true for thumb reconstruction. Soft tissue coverage of a thumb defect should be durable, pliable enough not to impede mobility, maintain length, and ideally restore at the minimum protective sensation.^1^

Reconstructive options for soft tissue coverage of a distal dorsal thumb defect include the following: skin graft (though grafting over exposed bone is commonly results in an unstable closure prone to failure and break down secondary to pressure), reverse cross-finger flap, modified Moberg (palmar advancement) flap, first dorsal metacarpal artery flap, and the Brunelli flap, the option chosen for coverage in this case.

The Brunelli flap is a homodigital, pedicled flap based on the dorsoulnar artery, a branch off the princeps pollicis artery after it branches from the radial artery. First described by Brunelli at the Institute de la Main in Paris, the dorsoulnar artery of the thumb has been shown in anatomic studies to be a reliable, longitudinal vessel. It can be used for coverage of both distal dorsal and volar thumb defects.^2^

The dorsoulnar artery was identified perioperatively with a handheld Doppler probe. The dorsal arcade was marked at the level of the proximal nail fold, and a second mark was made at the location of the palmar anastomosis of the ulnar palmar artery with the dorsoulnar artery. The dorsoulnar artery courses 1 cm ulnar to the median longitudinal axis of the thumb. The flap was elevated en bloc in a proximal to distal fashion while keeping the pedicle 1 cm in width so not to violate the underlying vasculature. At the Interphalangeal joint, the flap was rotated, inset, and sutured into place. A full-thickness skin graft from the forearm was harvested to cover exposed dermis at the site of pedicle rotation. Finally, to close the donor site, a rotational advancement flap was designed through a backcut along the first web space.

The Brunelli flap has many advantages. It utilizes the reliable dorsal arterial system of the thumb, and the technique is relatively simple, resulting in minimal scarring. Finally, as a homodigital flap, there is no tethering or morbidity to adjacent digits, allowing for early mobilization of the thumb. Disadvantages include poor sensation, less of an issue for coverage of dorsal defects, and risk of mild web space contracture. Recent reported modifications of the Brunelli flap include adipofascial extension of the proximal aspect of the pedicled flap and reconstruction of a composite skin and bony defect using an osteocutaneous flap based on the periosteal branches of the dorsoulnar artery to the first metacarpal neck.^3^^,^^4^

**Figure:**